# Cardiovascular changes after administration of aerosolized salbutamol in horses: five cases

**DOI:** 10.1186/s13028-014-0049-z

**Published:** 2014-08-14

**Authors:** Daniela Casoni, Claudia Spadavecchia, Chiara Adami

**Affiliations:** 1 Anaesthesiology and Pain Therapy Division, Department of Clinical Veterinary Sciences of the Vetsuisse Faculty of Berne, Länggassstrasse 124, Berne, CH-3012, Switzerland

**Keywords:** Salbutamol, Horse, Anesthesia, Cardiovascular side effects

## Abstract

Prevention and treatment of intraoperative hypoxemia in horses is difficult and both efficacy and safety of therapeutic maneuvers have to be taken into account. Inhaled salbutamol has been suggested as treatment of hypoxia in horses during general anesthesia, due to safety and ease of the technique. The present report describes the occurrence of clinically relevant unwanted cardiovascular effects (i.e. tachycardia and blood pressure modifications) in 5 horses undergoing general anesthesia in dorsal recumbency after salbutamol inhalation. Balanced anesthesia based on inhalation of isoflurane in oxygen or oxygen and air and continuous rate infusion (CRI) of lidocaine, romifidine, or combination of lidocaine and guaifenesine and ketamine was provided. Supportive measures were necessary to restore normal cardiovascular function in all horses but no long-term adverse effects were noticed in any of the cases.

## Background

Despite delivery of high-inspired oxygen concentration, suboptimal arterial partial pressure of oxygen (PaO_2_) and hypoxemia are frequently observed in horses undergoing general anesthesia [1]–[4]. In this context, hypoxemia, defined as a PaO_2_ lower than 60 mm Hg is secondary to considerable changes in ventilation and blood flow distribution in the equine lung, among which the most important is the development of a large intrapulmonary right-to-left vascular shunting [3],[5]. Dorsal recumbency is positively correlated with the incidence of hypoxaemia. Preventing or treating intraoperative hypoxaemia has proved difficult and no ideal technique is currently available in the clinical practice. In light of its supposed efficacy in improving gas exchange efficiency, clenbuterol, a β-adrenergic agonist, was administered intravenously with inconsistent [6],[7] effects. However, the administration of salbutamol aerosol in intubated horses with low PaO_2_ has been previously demonstrated effective [1]. Salbutamol is a non-catecholamine β-adrenergic agonist with prevalent selectivity for β_2_ receptors, used in human patients affected by asthma and in horses with recurrent airways diseases. Although it is well known that there is a large inter individual variation in response to β-adrenoreceptor ligands in vivo, the β_2_–selectivity in vitro is characterized by a higher intrinsic activity and higher affinity at β_2_ than at β_1_-adrenoreptors [8].

The mechanism of its beneficial action on improving PaO_2_ under anesthesia is still unclear. It has been suggested that the improvement of ventilation/perfusion (V/Q) mismatches is mediated by the opening of small airways in perfused lung regions, thereby allowing uptake of oxygen by blood in those areas [1]. Improvement of peripheral airflow obstruction has also been postulated in patients affected by primary pulmonary hypertension [9]; instead, no improvement of pulmonary oxygenation secondary to bronchodilator effect has been reported in clinically healthy horses [10]. The technique has the main advantage of being easy to learn, straightforward, non-invasive and safe. In case of systemic absorption, salbutamol effects on β_1_ and β_2_ receptors located extrabronchially, could lead to positive inotropy and chronotropy secondary to cardiac stimulation and peripheral muscular vasodilation. However, no tachycardia was observed in horses following salbutamol administration in clinical settings [1]. In experimental healthy horses in dorsal recumbency, a positive chronotropic effect without clinical relevance was temporarily recorded [11]. This case series reports the occurrence of clinically relevant cardiovascular effects i.e. tachycardia in 4 cases, and ventricular arrhythmia associated with systemic arterial hypotension in 1 case following the inhalation of salbutamol aerosol in anaesthetized horses in dorsal recumbency.

## Case presentations

### Anesthesia protocol and monitoring

Details relative to the anesthesia protocols for each patient are summarized in Table 1. After endotracheal intubation, inhalational anesthesia was maintained with isoflurane in oxygen or in oxygen and air (Table 1) through a circle system designed for large animal and connected to an adjustable time cycled ventilator (DHV 1000 Large Animal Ventilator, Surgivet®, Smiths Medical). A multi-parameter-anaesthetic monitor (Datex Ohmeda S/5, GE) was used in all the cases to continuously monitor invasive and non-invasive arterial blood pressure, base-apex lead electrocardiogram, partial saturation of oxygen (SpO_2_), end-tidal CO_2_ (EtCO_2_), inhaled and exhaled oxygen and isoflurane concentrations and for cases 4 and 5 spirometry pressure-volume loops. Arterial blood was sampled with a closed technique and the blood gas analysis was performed with Radiometer ABL 800 Flex, Radiometer Medical A/S, Denmark. Gas pressures were measured in mmHg and converted to kPa. Infusion of balanced crystalloid solution (Ringer Lactate) was provided all over the anesthetic at 10–20 ml kg^−1^ h^−1^. Dobutamine was infused at variable dose (Table 1) targeting a mean invasive arterial pressure (MAP) of 70 mmHg.

**Table 1:** Details of anaesthetic protocol for each patient

**Case details**	**Patient**	**Procedure**	**Premedication**	**Induction**	**Maintenance**	**Ventilation**	**Time of salbutamol inhalation**	**Et Isoflurane%**	**Dobutamine dose μg kg-**^**1**^ **min**^**−1**^
Case 1¥	14 year old, gelding Swiss Warm Blood 760 kg	Emergency explorative laparotomy	Romifidine^1^ 40 μg kg^−1^ IV	Ketamine^3^ 2.2 mg kg^−1^ IV	Isoflurane in oxygen	IPPV fr = 8–10 min^−1^ TV = 10 ml kg^−1^ I:E ratio: 1:2 Peak PAW =30 cmH_2_0 PEEP =5 cmH_2_O	T_0_ = 45	Et Iso 1.1%	Minimum0.22
			Butorphanol^2^ 50 μg kg^−1^ IV	Diazepam^4^ 100 mcg kg^−1^ IV	Lidocaine 2% (30 μg kg^−1^ min^−1^) CRI		T_1_ = 35		Maximum 0.5
									At salbutamol inhalation 0.5
Case 2¥	12 year old, mare Island horse 350 kg	Emergency explorative laparotomy	Romifidine^1^ 50 μg Kg^−1^ IV	Ketamine^3^ 2.2 mg Kg^−1^ IV	Isoflurane in oxygen and air	IPPV fr = 5 min^−1^ TV = 11.5 ml kg^−1^ I:E ratio: 1:2 Peak PAW =20 cmH_2_0	T_0_ = 55	Et Iso 1%	Minimum 0.5
			Butorphanol^2^ 50 μg kg^−1^ IV	Diazepam^4^ 50 mcg kg^−1^ IV	Lidocaine 2% (25 μg kg^−1^ min^−1^), ketamine^3^( 10 μg kg^−1^ min^−1^) and guafenesin^5^ (0.4 mg^−1^ kg^−1^ min^−^1) CRI		T_1_ = 40		Maximum 1.5
									At salbutamol inhalation 0.75
				Guaifenesine to effect IV					
Case 3*	6 month old, male Swiss warm blood 265 kg	Elective scrotal herniorraphy and castration	Acepromazine^6^ 30 μg Kg^−1^ IM	Ketamine^3^ 2.2 mg Kg^−1^ IV	Isoflurane in oxygen	Spontaneous breathing IPPV (after salbutamol administration) fr = 6 min^−1^ TV = 15 ml kg^−1^ I:E ratio: 1:2 Peak PAW =25 cmH_2_0	T_1_ = 45	Et Iso 1.1%	Minimum 0.6
			Romifidine^1^ 50 μg Kg^−1^ IV	Diazepam^4^ 100 mcg kg^−1^ IV	Romifidine (0.6 μg kg^−1^ min^−1^) CRI		T_0_ = 55		Maximum 1.6
			L- Methadone^7^ 50 μg Kg^−1^ IV	Guaifenesine to effect IV					At salbutamol inhalation 0.6
Case 4*	4 month old, male Polo Pony 200 kg	Elective humbelical herniorraphy	Acepromazine 30 μg kg^−1^ IM	Ketamine^3^ 2.2 mg kg^−1^IV	Isoflurane in oxygen and air	Spontaneous breathing; IPPV (before salbutamol administration) TV = 2200 ml fr = 7 min^−1^ TV = 13 ml kg^−1^ (2550 ml) I:E ratio: 1:2 Peak PAW =30 cmH_2_0	T_0_ = 35	Et Iso 1.3%	Minimum 0.8
			Romifidine^1^ 60 μg kg^−1^	Diazepam^4^ 100 mcg kg^−1^ IV			T_1_ = 28		Maximum 1.6
			Butorphanol^2^ 40 μg kg^−1^						At salbutamol inhalation 1.6
Case 5†	10 year old, gelding male Swiss warm blood 670 kg	Emergency carpus arthroscopy	Romifidine^1^ 60 μg kg^−1^ IV	Ketamine^3^ 2.2 mg Kg^−1^ IV	Isoflurane in oxygen and air	Spontaneous breathing TV =5600 ml	T_0_ = 85	Et Iso 1.1%	Minimum 0.12
			L- Methadone^7^ 50 μg kg^−1^ IV	Diazepam^4^ 100 mcg kg^−1^ IV	Romifidine (0.6 μg kg^−1^ min^−1^) CRI		T_1_ = 70		Maximum 0.5
									At salbutamol inhalation 0.12

*This patient was ventilated after hypoxia and hypercapnia were detected; ¥ these patients underwent mechanical ventilation from the beginning of inhalational anesthesia;† this patient was allowed to breath spontaneously during anesthesia; ^1^Romifidine, Sedivet ®, 10 mg/ml, Boehringer Ingelheim, Switzerland; ^2^Butorphanol, Morphasol ®10 mg/ml Graeub AGE, Switzerland; ^3^Ketamine, Narketan ®, 100 mg/ml, Vetoquinol Suisse; ^4^Diazepam, Valium® 5 mg/ml, Roche, Basel, Switzerland; ^5^Guaifenesine 12.5% solution (currently out of the market); ^6^Acetilpromazine, Prequillan ®10 mg/ml Arovet AG; ^7^ L-methadone/fenpipramide 50/2.5 μg kg^−1^ IV Polamivet ®, Intervet, Switzerland.

*T0* = Time between romifidine injection at the sedation and salbutamol inhalation. *T1* = Time between ketamine injection at the injection and salbutamol inhalation expressed in minutes.

### Salbutamol administration

**Figure 1:**
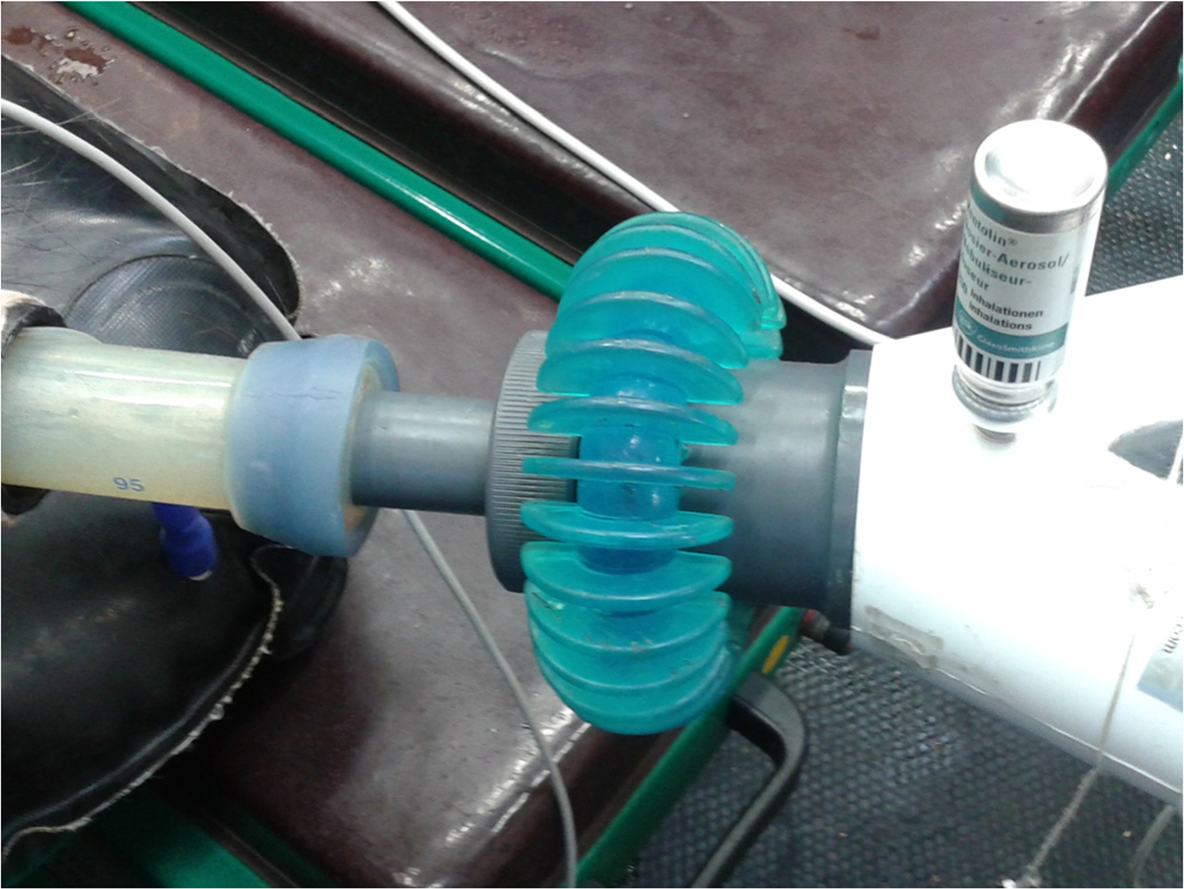
**Inhalation of salbutamol.** Salbutamol spray canister inserted into the adapter of the Y connection of the circle system during general anesthesia.

Salbutamol aerosol (Ventolin® nebulizer, 200 inhalations, Glaxo Smith Kline, Switzerland) as a racemic mixture was administered in all the cases for a total maximal dose of 2 μg kg^−1^. Salbutamol was sprayed through the same designed adapter into the Y-piece of the circle system (Figure 1) at the beginning of inspiratory phase for a number of respiratory acts equal to the number of nebulizations to be sprayed. Number of puffs to be administered was calculated for the weight of each patient. According to the manufacturer, each depression of the canister nozzle delivers 100 μg of active drug.

### Case 1

Twenty-five minutes after the beginning of inhalatory anesthesia, as arterial blood gas analysis revealed a PaO_2_ of 9.41 kPa (70.6 mmHg) (FiO_2_ = 0.92) salbutamol was administered. Mild hypercapnia (PaCO_2_ 6.97 kPa = 52.3 mmHg) and lactate concentration of 2.18 mmol/L were detected at the same time. Ten minutes following the administration, HR increased from 35 bpm to 48 bpm and remained elevated over 5 minutes. Concomitantly, profuse sweating and a decrease in systolic blood pressure without modification of the mean arterial pressure (MAP) (78 mmHg) were recorded. Heart rate (HR) returned to 34 bpm and concomitantly blood pressure increased as well and remained stable until the end of anesthesia. PaO_2_ increased after salbutamol administration and fifteen minutes later was 13 kPa (97 mmHg); PaCO_2_ was 7.33 kPa (55 mmHg) and lactate 2.3 mmol/L. Blood gas analysis was repeated 45 minutes after salbutamol inhalation and PaO2 decreased again to 79 mmHg. Since the PaCO_2_ increased concomitantly (8.08 kPa), mechanical ventilation was adapted. The horse recovered assisted uneventfully.

### Case 2

Thirty minutes after the beginning of inhalatory anesthesia, as arterial blood gas analysis revealed a PaO_2_ of 12.8 kPa (96 mmHg) (FiO_2_ = 0.72), salbutamol was administered. Normocapnia (PaCO_2_ 5.33 kPa = 40 mmHg) and lactate concentration of 3.08 mmol/L were detected at the same time. Ten minutes following the administration, HR increased from 46 bpm to 57 bpm and progressively reached the peak of 72 bpm (15 minutes later) to decrease over the following 10 minutes to 57 bpm and stabilize around 48 bpm subsequently. During the tachycardia phase, blood pressure did not change and remained low (MAP around 50 mmHg). Normal MAP could not be restored until HR decreased to the baseline, despite increasing of the dobutamine dose rate (up to a maximum of 1.5 μg kg^−1^ min^−1^) and the dose rate of fluid infusion. Concomitantly, profuse sweating was noticed. Fifteen minutes after salbutamol administration PaO_2_ increased to 26.67 kPa (200 mmHg); PaCO_2_ was 5.33 kPa (40 mmHg) and lactate 2.28 mmol/L. Blood gas analysis was repeated 60 minutes after salbutamol administration; PaO_2_ was 26.13 (196 mmHg) and PaCO_2_ was unmodified. The horse recovered assisted uneventfully.

### Case 3

Twenty minutes after the beginning of inhalatory anesthesia, the arterial blood gas analysis revealed a PaO_2_ of 14.26 kPa (107 mmHg) (FiO_2_ = 0.6). Ten minutes later salbutamol was administered. Moderate hypercapnia (PaCO_2_ 8.99 kPa = 67.4 mmHg) and lactate concentration of 1.9 mmol/L were detected at the same time. Five minutes following the administration, HR progressively increased from 40 bpm to 57 bpm over 15 minutes and then decrease over the next 10 minutes to baseline. A further mild decrease to a final HR of 38 bpm was recorded towards the end of anesthesia. During the tachycardia phase, blood pressure increased abruptly; the MAP passed from 63 to 105 mmHg, with a peak of diastolic blood pressure of 90 mmHg, followed by a progressive slow decrease to normal values. Concomitantly, the administration of dobutamine was interrupted. A new blood gas analysis conducted 20 minutes after the salbutamol administration revealed no improvement of PaO_2_ (14.26 kPa = 107 mmHg) and moderate hypercapnia was confirmed, therefore mechanical ventilation was started. The horse recovered assisted uneventfully.

### Case 4

Fifteen minutes after the beginning of inhalatory anesthesia, as arterial blood gas analysis revealed a PaCO_2_ of 10.04 kPa (75.3 mmHg) and a PaO_2_ of 9.34 kPa (70.5 mmHg) (FiO_2_ = 0.58), salbutamol was administered. A lactate concentration of 2.1 mmol/L was detected at the same time. Concomitantly IPPV was started. Measured tidal volume was 3.2 L. Spirometric volume- pressure loops indicated reduced compliance. Ten minutes after salbutamol administration, HR progressively increased from 42 bpm to 69 bpm and 15 minutes later reached the peak of 72 bpm to progressively decrease over the following 30 minutes to 57 bpm. A moderate decrease in the blood pressure was noticed after salbutamol administration and dobutamine was continuously administered over the entire procedure to maintain the MAP in the range of 69 to 72 mmHg. A new blood gas analysis conducted 30 minutes after salbutamol administration and beginning of IPPV revealed a clear improvement of PaO_2_ (37.9kPa = 284 mmHg); PaCO_2_ = 7.28 kPa and lactate 2.1 mmol/L. The horse recovered assisted uneventfully.

### Case 5

**Figure 2:**
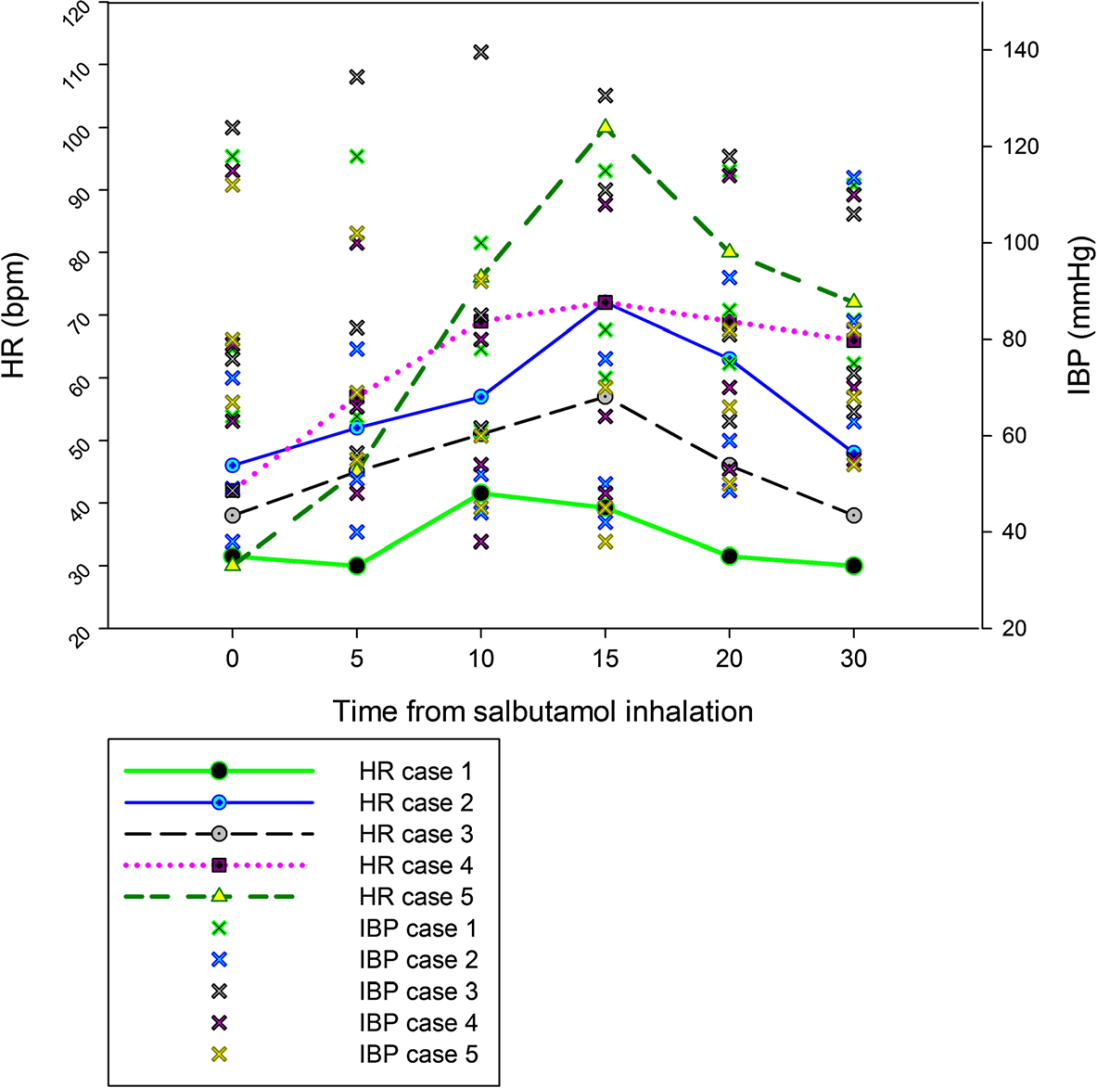
**Cardiovascular effect of salbutamol inhalation.** HR = heart rate (along the left y axis) and IBP = invasive blood pressure (along the right y axis) variations after salbutamol inhalation. On the x axis time 0 represents salbutamol inhalation. Systolic, mean and diastolic blood pressures are represented.

Fifteen minutes after the beginning of inhalatory anesthesia, arterial blood gas analysis revealed a PaCO_2_ of 7.04 kPa (52.8 mmHg) and a PaO_2_ of 13.65 kPa (102.4 mmHg) (FiO_2_ = 0.65). A second blood gas analysis was conducted 45 minutes later and revealed a PaCO_2_ of 7.49 kPa (56.2 mmHg) and a PaO_2_ of 10.18 kPa (76.4 mmHg) (FiO_2_ = 0.87). At this time point, salbutamol was administered. Five minutes after salbutamol administration, a profuse sweating started and HR progressively increased from 30 bpm to 46 bpm and in the following 15 minutes increased abruptly to 80 bpm and over ten further minutes reached the peak of 100 bpm. A run of premature ventricular complexes appeared on the sinus rhythm, followed by some regular sinus complexes, and immediately after ventricular tachycardia succeeded (recorded HR was between 95 and 100 bpm). Parallel with the development of tachycardia, the mean blood pressure decreased sharply from 80 to a minimum of 45 mmHg (Figure 2). Dobutamine CRI was stopped and a bolus of colloids of 2 ml kg^−1^ h^−1^ (Voluven® 6% balanced, Fresenius Kabi, CH) was administered over 10 minutes. A bolus of 1.5 mg kg^−1^lidocaine was administered over 5 minutes. Continuous rate infusion of phenylephrine was started at 0.01 μg kg^−1^ min^−1^ and then stopped when the MAP reached a value above 60 mmHg. Consequently, the HR decreased slowly and progressively and synusal rhythm reappeared. A new blood gas analysis conducted 20 minutes after salbutamol administration at FiO_2_ of 0.8 revealed ameliorated PaO_2_ (21.2 kPa = 159 mmHg) and moderate hypercapnia (PaCO_2_ = 8.44 kPa = 63.3 mmHg). Lactate plasma level (1.0 mmol/L) did not increase. After the end of the surgical procedure, which terminated 50 minutes after salbutamol administration, the horse was transferred to the recovery stall under ECG monitoring. Blood gas analysis at the end of the surgery was unvaried: PaO_2_ was 23 kPa (172.5 mmHg), PaCO_2_ 8 kPa (60 mmHg) and lactate plasma level 1.1 mmol/L. The trend of progressive, slow decrease of HR was confirmed and when the horse showed the first sign of nystagmus, romifidine at 10 μg kg^−1^ was administered as post-operative sedation and the ECG monitoring interrupted. The horse recovered assisted uneventfully.

## Conclusions

This case series describes the occurrence of adverse cardiovascular effects in 5 horses associated with the inhalation of salbutamol at clinical advised doses during general anesthesia. The treatment of suboptimal oxygen arterial pressure in horses has proven to be difficult and no efficacious therapy to counteract it has been established yet. The authors utilize in their daily practice inhalational salbutamol as treatment of hypoxemia taking advantage of the ease and the non-invasiveness of the technique. However, the occurrence of a cardiovascular impairment in the five cases described above warns against potential detrimental effects of the β-2 agonist inhalation. To the best of the authors’ knowledge there are no previous reports in literature describing the appearance of cardiovascular adverse effects after salbutamol administration in a clinical setting. In 4 out of 5 horses, the cardiovascular impairment could be corrected by adjusting the supportive therapy already administered during the anesthesia. However, in one horse (case 5) the occurrence of ventricular tachycardia and the severe decreasing of the blood pressure required additional therapy. As the peripheral vasodilation was a major concern and as the surgical operation was almost completed, the hypotension was treated with phenylephrine in light of its prevalent action as vasoconstrictor acting on post-synaptic α-1 receptors. In none of the cases was cardiac output measured before or after the salbutamol administration, therefore variations in blood pressure and heart rhythm were the only objective indicators of cardiovascular derangement. In light of the strong temporal association between the administration of salbutamol and the recorded cardiovascular impairment, the authors consider the inhalation of salbutamol its most likely cause. In none of the cases, was the sudden tachycardia either associated with surgical incision or further painful stimulation and supportive measures to restore the cardiovascular stability were successful. Hypovolemia was unlikely the case of the cardiovascular modification in case 1 as the observed tachycardia was of short duration and associated with sweating. In case 2, despite the suboptimal blood pressure recorded at the beginning of anesthesia could have been indicative of a fluid deficit, hypovolemia cannot explain the sudden tachycardia and the profuse sweating after inhalation of salbutamol. In case 5 no arrhythmias were detected prior or after the anaesthetic and no abnormalities were recorded on the ECG once the horse recovered, therefore a pre-existing cardiac disease seems to be unlikely in this horse. Investigating the clinical impact of such cardiac impairment on a longer term is difficult; variations of lactate serum levels after salbutamol administration could be indicative of impaired peripheral perfusion. The arterial lactate measured after salbutamol administration was not increased in our cases, however all the horses underwent supportive therapy which could have prevented the reduction of peripheral perfusion.

Cardiovascular impairment following inhalation of salbutamol is probably owing to systemic uptake.

In our cases, two anesthetists (DC and CA) used the same technique to spray salbutamol into the trachea as previously described. This technique has been described by Robertson and Bailey [1] in clinical horses lying in dorsal recumbency and utilized by Patschova *et al*. [11] in experimental horses.

In our cases, the total number of nebulizations to be administered was calculated on the base of measured weight. Therefore is improbable that an absolute over-dosage due to any procedural change could be the cause of the observed adverse effects. However, different filling of the canister, different humidity degree of the inner part of the inspiratory limb and endotracheal tube and slightly different delivered flow of the carrier gases cannot be ruled out as factors influencing the actual dose of drug delivered. Whether these potential differences in the actual dose could have influenced the percentage of systemic absorption is difficult to evaluate. More importantly, we did not deliver salbutamol through the MDI adapter (PriMed Co, Largo, Florida, USA) that the previous authors [1],[8] utilized. This adapter is specifically designed to allow salbutamol entering the midstream of gas flow and carrying it far down the bronchial tree allowing its binding to the target sites; thus, in our case, salbutamol could have precipitated earlier, leading to partial absorption into the systemic blood stream from the trachea. If so, a better standardization of the delivery technique has to be taken into higher consideration and alternative administration techniques [12] only consciously advised.

Independently of the adapter, the hypothesis of a clinical relevant systemic uptake of the drug was already formulated by Robertson and Bailey, as they noticed an excessive sweating in some horses, but not accompanied by an increase in heart rate. Inhaled β_2_ adrenoreceptor agonists, to which salbutamol belongs, are the most effective known bronchodilators. It has been demonstrated that inhaled salbutamol induces acute cardiac electrophysiologic effects in humans, especially tachycardia and shortened node recovery time [13]. In human medicine there is a body of evidence that β_2_ receptors are not only represented into the tracheal and bronchial musculature, but also in the atrium and ventricular myocardium and play a significant role in physiologic and pathologic conditions [14],[15]. It is well known that the equine tracheal musculature expresses predominantly β_2_ receptors [16]; however contrasting results are reported concerning their concentration in the heart. Indeed, Törneke [17] found a small proportion of β_2_ adrenoreceptors in the normal equine ventricular myocardium, while Horn *et al*. [18] could not demonstrate the presence of β_2_ adrenoreceptors in normal equine myocardium, and concluded that their density increases only during heart failure. According to the same authors, this discrepancy between studies could be attributed to differences among individual horses or between sites of ventricular muscle sampled. It is also difficult to find evidence whether the prevalent selectivity towards β_2_ receptors reported for salbutamol is relevant “in vivo”. It can be hypothesized that in some horses salbutamol could have acted as β_1_ agonist as well. Therefore, the systemically absorbed salbutamol could have caused the chronotropic effects via β_1_ adrenoreceptor activity, which are anyhow the prevalent population in the equine heart. Alternatively, one could speculate that horses with greater population of β_2_ receptors could be more sensitive to the action of salbutamol than horses with a smaller population.

Differently from what could be expected, the blood pressure trend recorded concomitantly to the rhythm modification in our 5 cases was inconsistent. Although the specific mechanisms that control vascular tone are not completely understood, it has been shown in rats that increasing density of β_2_ receptors in the vascular walls enhances vasorelaxation [19]. Different anaesthetic protocols were used in the described cases. However, in all cases, romifidine was administered as premedication and in case 3 and 5 additionally as CRI, salbutamol being administered relatively early after the beginning of inhalation anesthesia. Romifidine infused at the dose of 40 μg kg^−1^ h^−1^ has been reported not having influence on the blood pressure in isoflurane anaesthetized horses premedicated with romifidine at a standard dose of 80 μg kg^−1^[20]. However, it cannot be excluded that the hypotension observed in the case 1 and 4 was brought about by the activation of the vascular β_2_ receptors, which overcame the increased vascular resistance mediated by the action of romifidine administered in premedication on the α_1_ receptors. A decrease in stroke volume owing to an excessive reduction of diastolic time should also be taken into account, especially in case 4, in which the tachycardia was major. In case 5, lidocaine and phenylephrine were efficacious in restoring blood pressure and normal cardiac rhythm: abnormal ventricular contraction or massive vascular relaxation can both be claimed as responsible of the symptoms. Whether it was the normalized cardiac contractility or the improved vascular tone, the main contributor to the clinical amelioration cannot be differentiated.

In two cases (case 3 and case 5) the patients received salbutamol while they were breathing spontaneously. The decision to administer inhalational salbutamol as a corrective measure of inadequate PaO_2_ instead of starting IPPV was based on the values of PaCO_2_ measured in the blood. In the cases of normocapnia or slight hypercapnia (PaCO_2_ up to 55 mmHg) the preference of the authors was to allow the patients to breath spontaneously. Although the difference between the amount of drug delivered through a negative and a positive pressure driven inspiratory phase has never been tested, it is unlikely that a lower tidal volume can be correlated with a higher percentage of inspired salbutamol after a non-preset inspiratory act. In case 3, the spirometry was not included in the monitoring, but in case 5 a tidal volume of 5.6 L was recorded from the Pitot tube and this volume is lower than the volume one would have set in a regimen of mechanical ventilation. Therefore it is unlikely that the spontaneous breathing could have been the cause of increased inspired salbutamol. However it is worth mentioning that the inhalation of salbutamol has been previously described in horses under controlled ventilation, which implies a different respiratory mechanics from spontaneous breathing. When considering either the efficacy of the technique or the collateral effects observed, this variable has also to be taken into account.

It is also difficult to define which cut-off of PaO_2_ should be chosen to plan a salbutamol treatment. In fact, due to the great affinity of equine hemoglobin for oxygen, absolute hypoxemia is arbitrarily defined as a PaO_2_ lower than 60 mmHg [12],[21]. However, suboptimal PaO_2_ reflecting an inadequacy of ventilation can be identified in horses during general anesthesia and still represents a therapeutic challenge. PaO_2_ might even not improve, as observed in case 3, if inhalation of salbutamol is provided at higher PaO_2_ without addressing other potential causes of hypoxia. Furthermore, if the improvement of the PaO_2_ is not followed by an increase in the oxygen delivery, due to harmful cardiovascular alterations, the therapeutic intervention may be useless or even harmful. Since the consequences of relative and absolute hypoxia in equine anesthesia are still not completely understood, and seem not be major [22], in the light of the potential complications observed, it is probably worth limiting the use of salbutamol to the cases of true hypoxia non-responsive to mechanical ventilation.

In conclusion, taking into account that significant cardiovascular effects of inhaled salbutamol can occur in horses as reported in humans, the use of inhaled salbutamol should not be considered completely harmless and its use has to be pondered. Other therapeutic options to improve oxygenation should be considered in spontaneous breathing horses. If the case, supportive therapy is required and crystalloids, dobutamine, phenylephrine and lidocaine can be administered for the treatments of salbutamol side effects. No further adverse consequences were either recorded until the end of general anesthesia or during the recovery phase.

## Consent

Written informed consent was obtained from the patients’ owners for the publication of this report and any accompanying images.

## Competing interests

The authors declare that they have no competing interests.

## Authors’ contributions

DC and CA carried the anesthesias out and were responsible of perioperative care in the five cases. They contributed at the same extent to the preparation of the manuscript. CS supervised the anesthetics and gave valuable input to the final manuscript. All authors read and approved the final manuscript.
